# ITM2A as a Tumor Suppressor and Its Correlation With PD-L1 in Breast Cancer

**DOI:** 10.3389/fonc.2020.581733

**Published:** 2021-02-12

**Authors:** Rui Zhang, Tao Xu, Yu Xia, Zhi Wang, Xingrui Li, Wen Chen

**Affiliations:** ^1^ Department of Thyroid and Breast Surgery, Wuhan No.1 Hospital, Wuhan, China; ^2^ Department of Thyroid and Breast Surgery, Tongji Hospital, Tongji Medical College, Huazhong University of Science and Technology, Wuhan, China; ^3^ Department of Obstetrics and Gynecology, Tongji Hospital, Tongji Medical College, Huazhong University of Science and Technology, Wuhan, China

**Keywords:** ITM2A, breast cancer, prognosis, PD-L1, immune infiltration

## Abstract

**Background:**

High expression of integral membrane protein 2A (ITM2A) was reported to be associated with favorable prognosis in several solid tumors including breast cancer. This study aimed to investigate the role of ITM2A in breast cancer, especially in respect to tumor microenvironment.

**Methods:**

ITM2A expression was evaluated based on qRT-PCR results on breast cancer specimens, as well as TCGA and GEO datasets. The influence of ITM2A expression on breast cancer cell proliferation and tumor growth were evaluated by CCK-8 assay, clonogenic assay, and murine xenograft models. Transwell assay was performed to observe the changes of invasion and migration capacity in breast cancer cells. To determine the biological functions of ITM2A, differentially expressed genes (DEGs) were screened based on RNA-sequencing data of MCF-7 cells overexpressed ITM2A. Then, functional annotation on DEGs was given by Gene Ontology and KEGG analysis. The stimulation on programmed cell death ligand 1 (PD-L1) expression when ITM2A overexpressed was determined by flow cytometry. Meanwhile, the correlation on expression levels between PD-L1 and ITM2A was tested *via* qRT-PCR on 24 breast cancer tissues, as well as public database.

**Results:**

We demonstrated that ITM2A was frequently downregulated in breast cancer. Patients with high expression levels of ITM2A had longer overall survival and relapse free survival. Overexpression of ITM2A inhibited proliferation and impaired cells capacity of invasion and migration *in vitro* and *in vivo*. The DEGs in breast cancer cells overexpressed ITM2A were found to be associated with immunity responses. Moreover, ITM2A was found to facilitate breast cancer cells to express PD-L1. The correlation between PD-L1 and ITM2A was verified with both qRT-PCR assay and public database. Additionally, it was found that breast cancer had higher ITM2A expression frequently had more tumor-infiltrating lymphocytes (TILs).

**Conclusion:**

In summary, we found that high expression of ITM2A reduced the aggressivity of breast cancer cells and had a favorable effect on outcomes of patients with breast cancer. Moreover, ITM2A induced PD-L1 expression in breast cancer cells was accompanied with higher TILs numbers in tumor microenvironment.

## Introduction

Integral membrane protein 2A (ITM2A) belongs to the Type II Integral Membrane protein (ITM2) family, along with ITM2B and ITM2C (1). This family belongs to the BRICHOS superfamily. ITM2A is preferentially expressed in T lineage cells among hematopoietic cells (2). Now we concentrate on the biological functions that ITM2A performs in breast cancer.

Breast cancer is frequently diagnosed among females globally. In the United States, it is estimated that 281,550 new cases of invasive breast cancer will be diagnosed in females in 2021 (3). Simultaneously an estimated 43,600 breast cancer deaths will occur. The treatments targeting breast cancer have been continually developed and advanced for more than 100 years. Those treatments include mastectomy, conserving surgery, endocrine therapy, and common anti-tumor regimens—chemotherapy and radiation therapy. Currently, the 5-year survival of patients with breast cancer is over 90% (4). Nevertheless, the survival of patients with triple-negative breast cancer (TNBC) is quite poor, resulting from lack of robust treatment strategies (5). The remarkably heterogeneous TNBC tumor microenvironment has added disadvantage to treatments. Whenever the clinical stage, chemotherapy is the primary established treatment option for patients with TNBC (5). In addition, metastatic breast cancer frequently has poor clinical outcomes with a 5-year survival rate at 26% (4). It is urgent to explore robust regiments to improve outcomes of patients with TNBC or metastatic breast cancer.

As a novel therapeutic approach that releases the brake on effector T cells activation, immune checkpoint blockade (ICB) therapy has achieved success in several solid malignancies. The most deeply investigated ICBs, including anti-cytotoxic T lymphocyte associated antigen 4 (CTLA-4), anti-programmed death 1 (PD-1), and anti-programmed death ligand 1 (PD-L1), are developed to bypass the immune checkpoint, with the aim of rescuing and enhancing the functions of antitumor T effector cells (6). Pembrolizumab is a representative reagent of anti-PD-1 monoclonal antibody. There was a phase 2 trial that demonstrated that women with high risk, stage II/III, ERBB2-negative breast cancer had improvement in pathological complete response (pCR) rate when pembrolizumab was added to standard neoadjuvant chemotherapy over patients who received chemotherapy alone (7). Pembrolizumab also has showed durable antitumor activity as first-line therapy for patients with PD-L1-positive metastatic TNBC (mTNBC) (8), as well as demonstrated durable antitumor activity in a subset of patients with previously treated mTNBC (9). Currently, a phase 3 clinical trial showed that among patients with stage II/III TNBC, patients who received pembrolizumab plus neoadjuvant chemotherapy had a significantly higher rate of pCR than those who received placebo plus neoadjuvant chemotherapy (10). Normally, the response rate in patients with other solid tumors when receiving anti-PD-1/PD-L1 antibodies hangs on multifactorial parameters, including PD-L1 expression, tumor mutational load/microsatellites status, and intensity of intratumoural CD8+ cytotoxic T cells (11–13). In breast cancer, PD-L1 expressed on the surface of tumor cells as well as infiltrating lymphocytes (14). Meanwhile, PD-L1 expression associated with tumor-infiltrating lymphocytes (TILs) was found to be a positive prognostic feature in breast cancer (14, 15).

In this study, we explored if ITM2A could influence the proliferation and aggressivity of breast cancer cells. Meanwhile, RNA-sequencing (RNA-seq) of breast cancer cells that overexpressed ITM2A was conducted. We found ITM2A was associated with immunity responses. More importantly, ITM2A was found to induce PD-L1 expression as well as be associated with TILs.

## Methods

### Public Database Analysis

For comparing ITM2A expression between breast cancer samples and normal samples, gene expression profiles (GSE29413 containing 12 normal tissues and 54 breast cancer samples; GSE61304 containing 4 normal tissues and 58 breast cancer tissues) were downloaded from the Gene Expression Omnibus (GEO) database (https://www.ncbi.nlm.nih.gov/geo/). In addition, gene expression date of n = 110 breast cancer tissues and paired normal tissues were selected from TCGA (https://portal.gdc.cancer.gov/). Tumor Immune Estimation Resource (TIMER; https://cistrome.shinyapps.io/timer/) was utilized to exam the correlation between ITM2A expression and TILs (16).

### Specimens Collection and Processed

Human tumor samples were obtained from patients diagnosed with invasive breast cancer who had not received neoadjuvant chemotherapy. A total of 24 breast cancer tissues and paired adjacent tissues were obtained during surgery at the Department of Thyroid and Breast Surgery, Tongji Hospital, Tongji Medical College of HUST. Specimens were immersed in cold RNA later solution overnight and then stored at -80°C. Each informed consent was signed by patients and approval of experiment on human specimens was received from the Ethics Committee of Tongji Hospital.

### Survival Analysis

For survival analysis, the online tools—Kaplan-Meier Plotter (http://kmplot.com/) (17) and PrognoScan (http://www.prognoscan.org/) (18)—were used to detected the overall survival (OS), progression free survival (PFS), and distant metastasis free survival (DMFS) of patients grouped by the median of ITM2A mRNA.

### Cell Culture

MCF-7 and MDA-MB-231 cells were obtained from the Chinese Academy of Science cell bank (Wuhan, China). The cell lines were authenticated by using short tandem repeat (STR) DNA profiling (ABI 3730XL Genetic Analyzer, Life Technologies, Waltham, MA, USA). MCF-7 cells were cultured in DMEM supplemented with 10% FBS and 1% penicillin/streptomycin (Invitrogen, USA) at 37°C with 5% CO_2_. MDA-MB-231 cells were cultured in L-15 (HyClone, USA) supplemented with 10% fetal bovine serum (FBS) and 1% penicillin/streptomycin (Invitrogen, USA) at 37°C. These cells passaged less than 30 times during our experiment.

### Transfection

The MCF-7 and MDA-MB-231 cells were grown in 6-well plate to arrive at 50%–60% or 70%–80% confluence respectively. Then cells were transfected with 2.0 ug empty vector or ITM2A plasmid using X-tremeGENE HP DNA Transfection Reagent (Roche, USA) according to the manufacturer’s instructions. After transfection for 48 h, cells were harvested or passaged for subsequent experiments.

### Quantitative Real-Time PCR

Total RNA was extracted from tissues or cell pellets using Trizol reagent (Invitrogen, USA) and reversely transcribed using PrimeScript RT Reagent Kit (Takara Bio Inc.). The expression levels of interested genes were measured in triplicate using SYBR Green qPCR Mix (Toyobo, China). Primer sequences were as follows. ITM2A Forward: 5´-ATCCTGCAAATTCCCTTCGTG-3´, Reverse: 5´- CAGGTAAGCAGTCATTCCCTTT-3´; PD-L1 Forward: 5´- CTGTCACGGTTCCCAAGGAC -3´, Reverse: 5´-GGTCTTCCTCTCCATGCACAA-3´; GAPDH Forward: 5´- CTCACCGGATGCACC AATGTT -3´ Reverse: 5´-CGCGTTGCTCACAATGTTCAT -3´. Relative mRNA expression was calculated using the 2^-ΔΔCT^ method, and GAPDH was used as an internal control (19).

### Immunoblotting Analysis

For immunoblotting, cells were lysed in iced RIPA buffer supplemented with protease and phosphatase inhibitors (Roche, USA). After centrifuging in high speed, the lysates were purified and then were separated in 5–12% SDS-PAGE gels. The lysates were transferred to PVDF membranes and blocked for 1 hour with 5% bevor serum albumin (BSA). The primary antibodies we used were as follows: anti-ITM2A (Proteintech, Wuhan, China); anti-α-Tubulin (Proteintech, Wuhan, China). Membranes were incubated with a species-matched HRP-conjugated secondary antibody for 1 h. α-Tubulin was used as loading controls to quantify the results.

### Clonogenic Assay

For clonogenic assay, cell lines transduced with empty vector or ITM2A were seeded in 6-well plates (1,000 cells per well), typically for 14 days. Cells were fixed with 4% formaldehyde for 15 min followed by crystal violet staining for 15 min.

### Cell Counting Kit-8 (CCK-8) Assay

Cell viability was determined using CCK-8 (DOJINDO, Japan) assay. Briefly speaking, cells transfected with indicated plasmid were plated in 96-well plates and cultured for 24–72 hours. Then the medium in each well was replaced with 100 ul fresh culture medium supplemented with 10 ul CCK-8. After incubation for 2 hours at room temperature in the dark, the absorbance at 450 nm wave length was read by microplate reader (Bio-Rad, USA). Cell viability was evaluated based on the absorbance value relative to wells plating no cells.

### Transwell Migration and Invasion Assay

Migration and invasion assay were performed according to the manufacturer’s instructions (Corning, USA). Briefly, about 5 × 10^4^ MDA-MB-231 cells transfected with empty vector or ITM2A plasmid were plated into upper chamber coated with 200 ul L-15 with 10% FBS. In the lower chamber, 500 ul medium with 40% FBS was used as a chemotactic agent. Nineteen hours later, the insert was removed. Cells in the microporous membrane were washed with PBS and then stained with crystal violet at room temperature for 10–15 min. As invasion assay, the upper chamber was coated with Matrigel besides medium and 10% FBS, and the incubation time was 36 hours. Cells in the microporous membrane were counted in five random fields per chamber.

### Flow Cytometric Analysis

The FITC Annexin V Apoptosis Detection Kit I (BD Biosciences, USA) was used to detect apoptosis rate of cells. The collected fresh cell pellets were washed using cold PBS and then suspended by 1 × binding buffer. Next, 5 ul of Annexin V and 5 ul of PI was added into 300 ul of 1 × binding buffer contained around 1 × 10^5^ cells. Cells were gently mixed and then incubated for 15 min at room temperature in the dark. Then cells were analyzed on the FACS Calibur System (Beckman Coulter). The PE Mouse Anti-Human PD-L1 (Cat 329706; BioLegend, Inc.), APC Mouse Anti-Human PD-L2 (Cat 345507; BioLegend, Inc.), and PerCP/Cyanine5.5 Mouse Anti-Human B7-H3 (Cat 351009; BioLegend, Inc.) were used to detect PD-L1, PD-L2, and B7-H3 in MCF-7 and MDA-MB-213 cells. First, fresh cell pellets were collected and washed with PBS, then were suspended in 300 ul of PBS containing 1‰ BSA and incubated with corresponding antibody for 30 min on ice in the dark. Finally, staining cells were washed and then analyzed on FACS Calibur System (Beckman Coulter). FlowJo (ver. 10.0) was used for data acquisition and analysis.

### Mouse Xenograft Studies

Female BALB/c null mice between the ages of 4–6 weeks were purchased from the Beijing HFK Bio-Technology Co., Ltd. The mice were bred in a specific-pathogen free facility. Prepared MCF-7 cells were inoculated subcutaneously under axilla (1 × 10^6^ cells per mouse). Once the tumors were tangible, its volume was calculated using the formula 0.5 × (minor tumor axis)^2^ × (major tumor axis) once in 3 days. Before the nude mice were sacrificed, magnetic resonance imaging examination (plain scan and enhanced) was conducted to check the growth of tumors in the body. All animal procedures were performed in accordance with the approved Guide for the Care and Treatment of Laboratory Animals of Tongji Hospital and approved by the Ethics Committees of Tongji Hospital.

### RNA Sequencing on MCF-7 Cells and DEGs Screening

MCF-7 cells were transfected with 2.0 ug empty vector or ITM2A plasmid as above described. Three days after transfection, the MCF-7 cells were collected using trypsin (Cat 0458-250G; Lifescience). Then total RNA was derived from cells pellets using Trizol (Invitrogen, USA) according to the manufacturer’s protocol. A total amount of 2 ug RNA per samples was sent to BerryGenomics (Beijing, China) for next generation sequencing. Briefly, mRNA was purified from total RNA and cDNA was synthesized. Then the 3’ ends of DNA fragments were adenylated and adaptor with hairpin loop structure were ligated. After PCR, the libraries were sequenced on an Illumina NovaSeq platform to generate 150 bp paired-end reads, according to the manufacturer’s instructions. The DEGs between ITM2A overexpressed cells and nature control cells were identified using the DEGseq R package according to below criterions: the adjusted p < 0.05 and |log2-fold-change| > 2.

### Functional Annotation and Enrichment Analysis

Gene Set Enrichment Analysis (GSEA) was performed on RNA-Seq profiles of 1,053 breast cancer stratified by ITM2A mRNA expression levels using GSEA software as previously described (20). For DEGs derived from ITM2A overexpressed cells, the R packages “clusterProfiler” and “enrichplot” were used to conduct Gene Ontology (GO) function and Encyclopedia of Genes and Genomes (KEGG) pathway enrichment analysis (21). It was considered statistically significant when the adjusted p < 0.05.

### Statistical Analysis

Statistics analysis in this experiment was conducted by SPSS 22.0 and GraphPad 8.0. Comparisons between two groups were determined by two tailed Student’s t-test or chi-square test. The data were expressed as mean ± standard deviation from at least three independent experiments. The correlation of gene expression was accessed by Pearson’s correlation coefficient. *P*-value < 0.05 was taken as statistically significant and “ns” represented *P*-value ≥ 0.05; “*” represented *P*-value < 0.05, and “**” represented *P*-value < 0.01.

## Result

### ITM2A Was Decreased in Breast Cancer and Positively Associated With Favorable Survival

**Figure 1:**
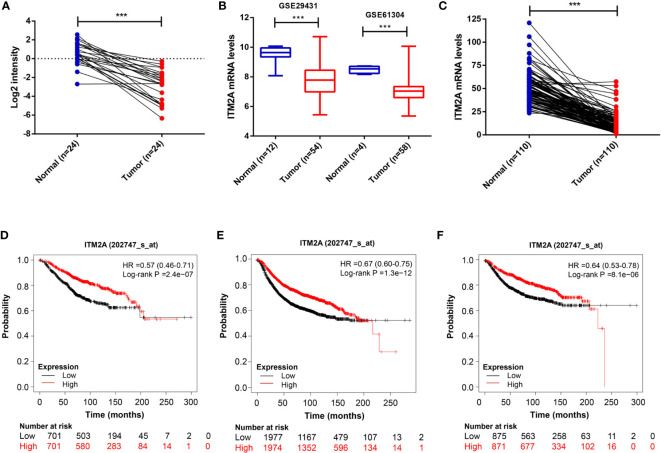
ITM2A was down-regulated in breast cancer and positively associated with favorable outcomes. **(A)** Comparison of ITM2A expression in breast cancer tissues with that of paired adjacent normal tissues. **(B)** Boxplot showing expression level of ITM2A in cancer tissues and normal tissues in the breast profile GSE29431 and GSE61304. **(C)** Comparison of ITM2A expression in cancer tissues with that of paired adjacent normal tissues in the breast TCGA dataset. **(D-F)** Kaplan-Meier survival curves depicting the OS **(D)**, RFS **(E)**, and DMFS **(F)** of patients with breast cancer stratified by ITM2A mRNA levels. ***p < 0.001. OS, overall survival; RFS, relapse free survival; DMFS, distant metastasis free survival.

First, we analyzed ITM2A expression in collected specimens and found that ITM2A was decreased in breast cancer compared to normal tissues (**Figure 1A**). The down regulation of ITM2A in breast cancer tissues was further verified in GEO and TCGA datasets (**Figures 1B, C**). This result was square with the research performed by Cefan Zhou et al. (22). Additionally, ITM2A was observed to be decreased in acute myeloid leukemia (23), cervical cancer (24), and ovarian cancer (25). Based on the TIMER database, there were more than 10 types of cancer with lower expression of ITM2A (**Supplementary Figure 1**). We then evaluated the prognostic value of ITM2A mRNA in patients with breast cancer. It was proved that patients with increased ITM2A had longer overall survival (OS) (HR = 0.57 [0.46–0.71], Log-rank P = 2.4×10^-7^) (**Figure 1D**), disease free survival (DFS) (HR = 0.67 [0.60–0.75], Log-rank P = 1.3×10^-12^) (**Figure 1E**), and distant metastasis free survival (DMFS) (HR = 0.64 [0.53–0.78], Log-rank P = 8.1×10^-6^) (**Figure 1F**). PrognoScan is a database for met-analysis of the prognostic value of genes (26). In PrognoScan, high expression of ITM2A in BC was found to be associated with longer relapse free survival (RFS), disease specific survival (DSS), as well as OS and DMFS (**Table 1**). Collectively, we found that ITM2A was positively associated with favorable survival.

**Table 1:** Survival analysis of ITM2A mRNA in breast cancer patients (the PrognoScan database).

Dataset	Endpoint	Probe ID	Number	COX P-value	HR [95% CI-low CI-up]
GSE19615	DMFS	202747_s_at	115	0.0342	0.39 [0.16–0.93]
GSE19615	DMFS	202746_at	115	0.0166	0.43 [0.22–0.86]
GSE11121	DMFS	202747_s_at	200	0.0370	0.70 [0.51–0.98]
GSE11121	DMFS	202746_at	200	0.0080	0.57 [0.37–0.86]
GSE2034	DMFS	202747_s_at	286	0.0042	0.75 [0.62–0.91]
GSE2034	DMFS	202746_at	286	0.0241	0.75 [0.58–0.96]
GSE1456	RFS	202747_s_at	159	0.0050	0.66 [0.49–0.88]
GSE1456	OS	202746_at	159	0.0005	0.40 [0.26–0.62]
GSE1456	DSS	202747_s_at	159	0.0031	0.60 [0.43–0.84]
GSE1456	OS	202747_s_at	159	0.0017	0.63 [0.47–0.84]
GSE1456	RFS	202746_at	159	0.0001	0.43 [0.28–0.66]
GSE1456	DSS	202746_at	159	0.0001	0.37 [0.22–0.62]
GSE3494	DSS	202746_at	236	0.0361	0.68 [0.47–0.98]
GSE3494	DSS	202747_s_at	236	0.0266	0.74 [0.57–0.97]
GSE49226	DFS	202747_s_at	249	0.0433	0.80 [0.65–0.99]

### ITM2A Inhibited Migration and Promoted Apoptosis of Breast Cancer Cells

**Figure 2:**
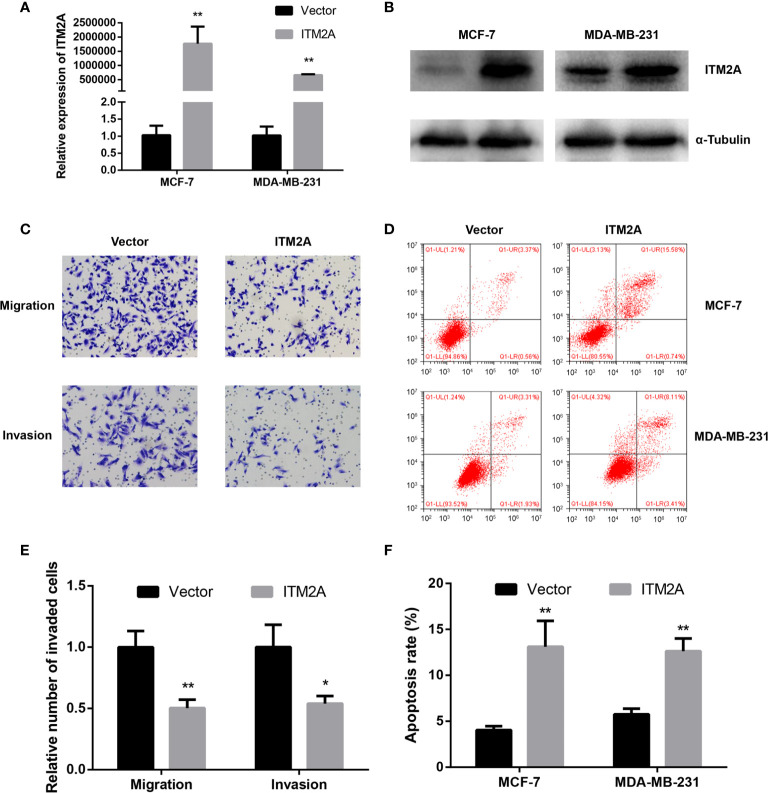
Overexpression of ITM2A inhibited migration and promoted apoptosis of breast cancer cells. MCF-7 and MAD-MB-231 cells were transfected with the indicated plasmid. qRT-PCR **(A)** and immunoblotting **(B)** were used to test ITM2A expression in mRNA and protein levels 48 hours after transfection. **(C, E)** Migration and invasion assay in MDA-MB-231 cells. **(D, F)** Apoptosis rate in MCF-7 and MDA-MB-231 cells 48 hours after transfection. *p < 0.05, **p < 0.01.

To get an in-depth understanding of the role of ITM2A in the tumorigenicity of breast cancer, plasmid encoding ITM2A (OE-ITM2A) or empty vector was transfected into two frequently used breast cancer cell lines: MCF-7 and MDA-MB-231 cells. Overexpression of ITM2A by OE-ITM2A transfection was validated by qRT-PCR (**Figure 2A**) and immunoblotting (**Figure 2B**). Meanwhile, ITM2A overexpressed cells had a reduced capacity to immigrate and invade (**Figures 2C, E**). Moreover, we observed a higher apoptosis rate in OE-ITM2A transfected cells than in empty vector transfected cells using FASC (**Figures 2D, F**). Our findings demonstrated that ITM2A could inhibit the migration and invasion of breast cancer cells. At the same time ITM2A had the ability to promote apoptosis in breast cancer cells.

### Overexpression of ITM2A Decreased the Proliferation of Breast Cancer In Vitro and In Vivo

**Figure 3:**
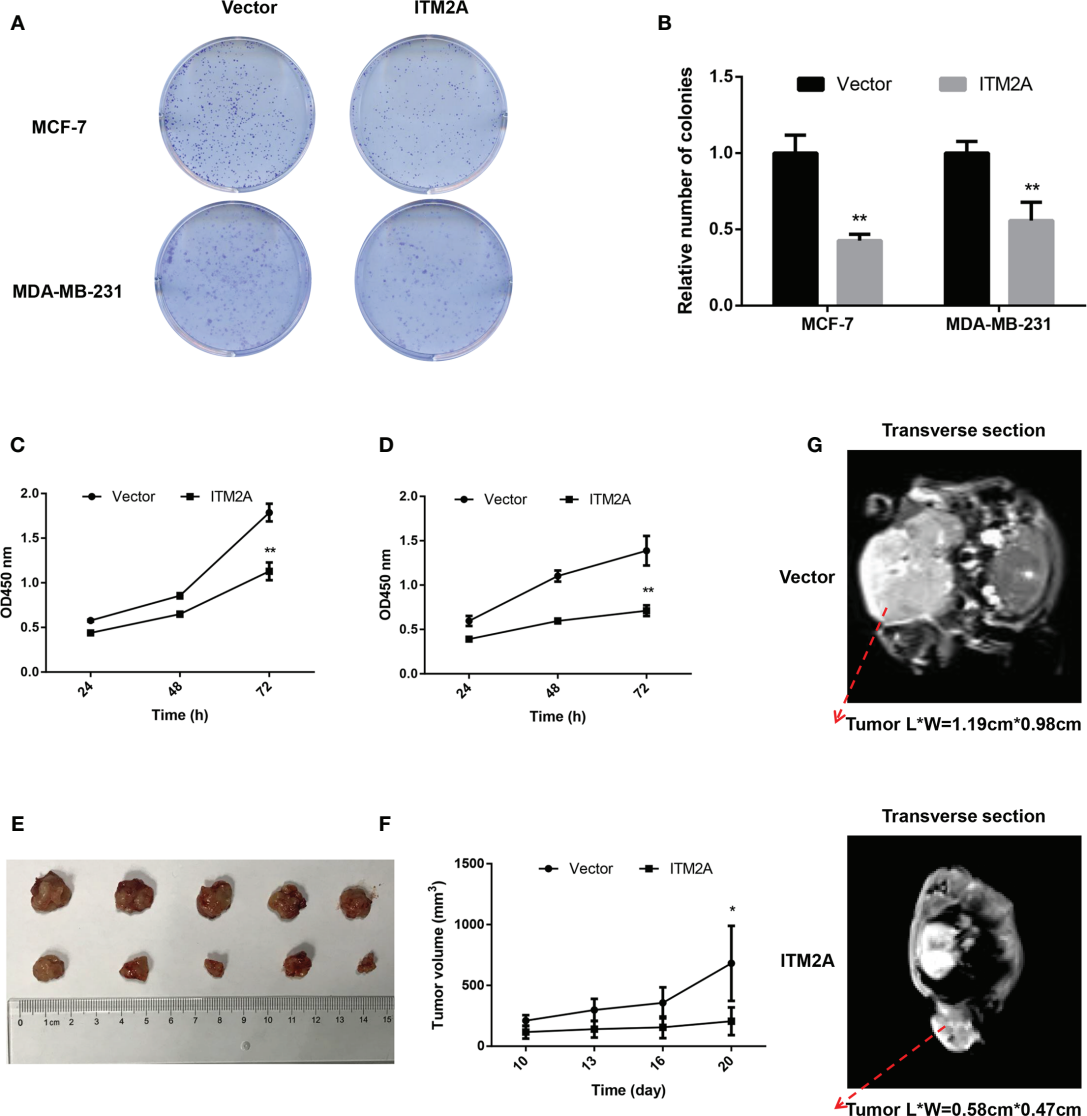
ITM2A overexpression decreased proliferation of breast cancer *in vitro* and vivo. **(A, B)** Clone formation was stained with crystal violet 14 days after seeded. **(C, D)** Growth curves of MCF-7 (left) and MDA-MB-231 cells (right) transfected with the indicated plasmid were measured by CCK-8 assay. **(E-G)** ITM2A expression inhibited the proliferation of breast cancer cells *in vivo*. **(E)** Photographs of dissected tumors from sacrificed mice. **(F)** Growth curves of indicated tumors in BALB/c null mice. **(G)** Representative images of enhanced MRI in transverse section. “L” represents the major tumor axis and “W” represents the minor tumor axis. *p < 0.05, **p < 0.01.

To validate the inhibitory proliferation in breast cancer cells with ITM2A overexpression in the long term, we then seeded cells transfected with plasmid into 6-well plates and counted the number of clones 14 days later. It was proved that both MCF-7 and MDA-MB-231 formed fewer clones when OE-ITM2A was transfected (**Figures 3A, B**). Meanwhile, cell viability assays showed that proliferation of breast cancer cells was significantly attenuated after OE-ITM2A transfection (**Figures 3C, D**) at 72 hours. To explore how the ITM2A expression influence the breast cancer growth *in vivo*, we then planted MCF-7 cells that transfected with OE-ITM2A or empty vector under axilla of female BALB/c null mice. Those mice did not receive any treatments and were sacrificed 20 days later. It was proved that ITM2A overexpressed tumors were notably smaller than ITM2A normally expressed tumor with respect to MRI test and tumor size (**Figures 3E–G**). These results, in summary, demonstrated that ITM2A could impair the proliferation of breast cancer *in vitro* and *in vivo*.

### Overexpression of ITM2A Induced Immunity Relate Responses

**Figure 4:**
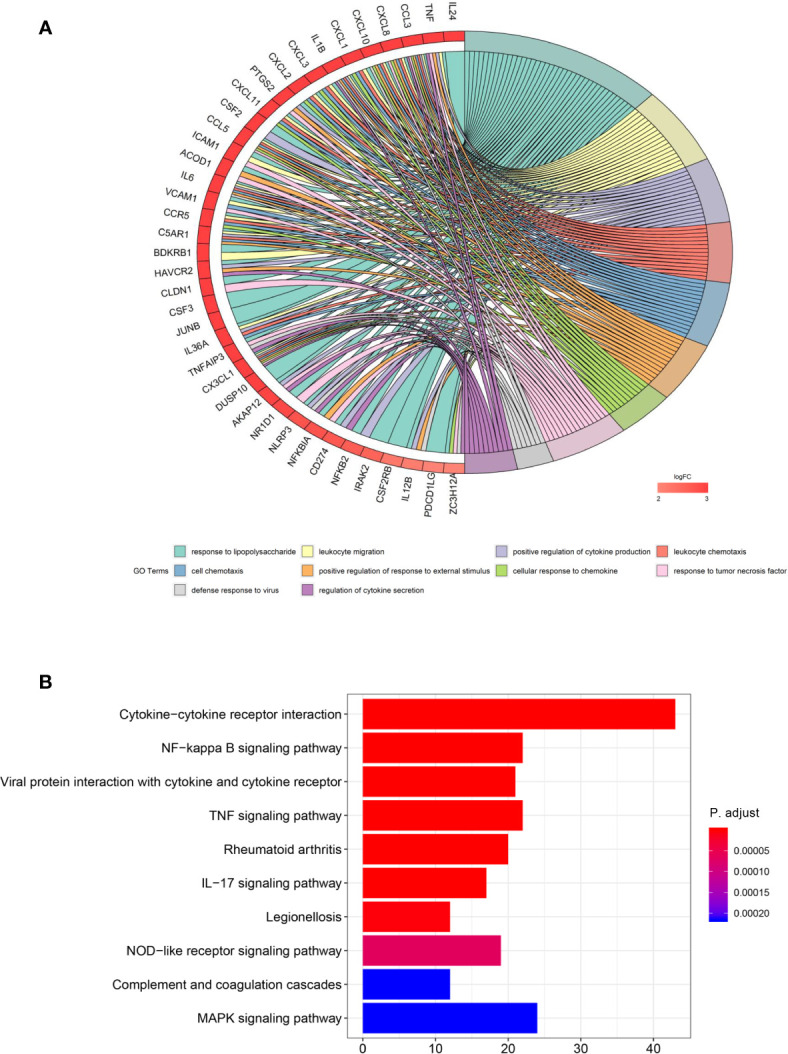
Overexpression of ITM2A induced immunity related response. **(A)** DEGs were derived based on RNA-seq data on MCF-7 cells that overexpressed ITM2A. Then the DEGs were mapped to GO terms and the top 10 ranked GO terms are showed. **(B)** The top-10 ranked KEGG pathways in which DEGs enriched. DEGs, differentially expressed genes; RNA-seq, RNA-sequencing; GO, gene ontology.

In order to understand the biological role of ITM2A in breast cancer cells, we performed RNA sequencing (RNA-seq) on MCF-7 cells that transfected with OE-ITM2A plasmid or vectors. The differentially expressed genes (DEGs) were derived (**Supplementary Figure 2**) and then mapped to GO terms and KEGG pathways. The most DEGs that map to GO terms were chemokine and interleukin, such as CCL3, CXCL8, IL24, and TNF (**Figure 4A**), which indicated a pro-inflammatory effects (27, 28). Consistent to these genes, the top ranked GO terms concentrated on cell chemotaxis (**Figure 4A**). When matched with KEGG pathway, the DEGs enriched in immunity related response (**Figure 4B**). The top three ranked pathways were cytokine-cytokine receptor interaction, NF-Kappa B signaling pathway, and viral protein interaction with cytokine and cytokine receptor. Additionally, GSEA analysis implied ITM2A was active in immunity related pathways (**Supplementary Figure 3**). To our knowledge the association between ITM2A and immunity response in breast cancer has never been reported. Thus, we focused on the role of ITM2A in breast cancer on context of immunity.

### ITM2A Increased PD-L1 Expression in Breast Cancer Cells

**Figure 5:**
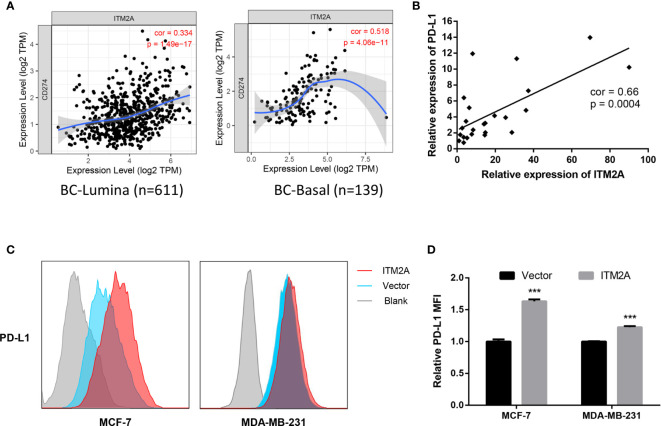
ITM2A increased PD-L1 expression in breast cancer cells. **(A)** Correlations between PD-L1 and ITM2A expression in lumina breast cancer (left) and basal breast cancer (right) based on TIMER database. **(B)** Correlation between PD-L1 and ITM2A expression in collected 24 breast cancer specimens. **(C, D)** MCF-7 and MDA-MB-231 cells were transfected with indicated plasmid. PD-L1 expression in these cells 48 hours after transfection was tested by flow cytometric analysis. ***p < 0.001.

Anti-PD-L1 therapy has been approved in TNBC treatment and PD-L1 expression levels were related to clinical response to these therapies (8). In addition, some evidence demonstrated other signaling roles of the PD-L1 molecule, including pro-survival, reducing mTOR activity, and glycolytic metabolism (29). We then explored if the ITM2A expression is associated with PD-L1 expression. Analysis based on the TIMER database revealed a positive correlation between ITM2A and PD-L1 in lumina and basal subtypes (**Figure 5A**). In addition, this correlation was verified by qRT-PCR, which quantified the relative expression levels of ITM2A and PD-L1 of 24 breast cancer specimens (**Figure 5B**). Furthermore, ITM2A could significantly upregulate the PD-L1 expression in both MCF-7 and MDA-MB-231 cells (**Figures 5C, D**), along with PD-L2 (**Supplementary Figures 4A, B**) and B7-H3 (**Supplementary Figures 4C, D**). PD-L2 is the second ligand for PD-1 and was reported to be associated with PD-L1 expression in melanoma, lung, and kidney cancer (30). Those results demonstrated that ITM2A could upregulate PD-L1, PD-L2, and B7-H3 expression in breast cancer cells.

### ITM2A Expression Was Positively Correlated With TILs Quantity

**Figure 6:**
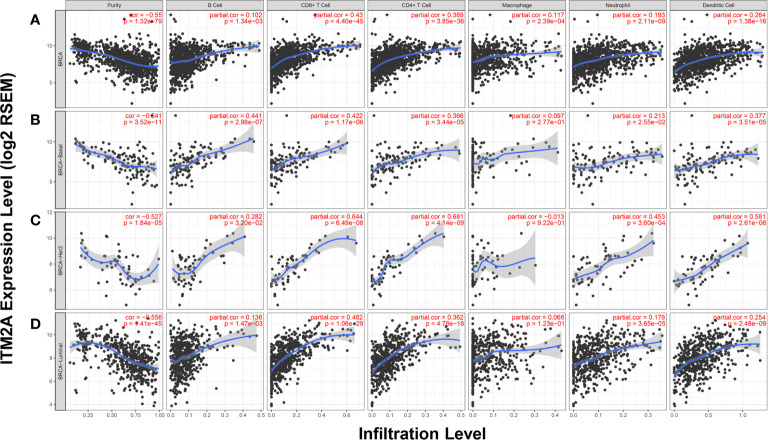
ITM2A expression was positively correlated with TILs quantity. Correlations between ITM2A expression and six TILs types in breast cancer were evaluated based on TIMER database. Analysis in all subtypes of BC **(A)**, basal **(B)**, HER-2 **(C)**, and luminal subtypes **(D)**.

The TILs have been considered as reliable biomarkers to predict breast cancer patients’ response to chemotherapy, as well as ICB (31, 32). Above results indicated the correlation between ITM2A and immunity response, as well as PD-L1 expression. We then examined if the ITM2A could predict the TILs intensity. The correlation between ITM2A expression and six TILs types (B cells, CD8+ cells, CD4+ cells, macrophage, neutrophil, and dendritic cell) were analyzed based on TIMER database. A positive correlation ranged from intermediate to high between ITM2A and CD8+ T cells was frequently observed over all subtypes of breast cancer (**Figures 6A–D**). The similar results were found in another five common cancer types, including lung adenocarcinoma (**Supplementary Figure 5A**), lung squamous cell carcinoma (**Supplementary Figure 5B**), colon adenocarcinoma (**Supplementary Figure 5C**), head and neck cancer (**Supplementary Figure 5D**), and prostate adenocarcinoma (**Supplementary Figure 4E**).

## Discussion

Before this study, there were a handful of studies that investigated the functions of ITM2A in tumor. A previous study found that ITM2A could inhibit ovarian cancer growth and induce G2/M cell cycle arrest, indicating that ITM2A was a novel tumor suppressor in ovarian cancer (25). Another study demonstrated that ITM2A inhibited breast cancer cells growth *via* enhancing autophagy induction through a mTOR-dependent manner (22). In this study, we explored the roles that ITM2A played in breast cancer. It was confirmed that ITM2A was decreased in breast cancer tissues. ITM2A inhibited breast cancer cells growth *in vitro* and *in vivo*, and reduced the aggressivity of breast cancer *via* impairing its immigratory and invading capacity. We also found that high expression of ITM2A was associated with a longer OS and RFS, etc. However, ITM2B or ITM2C expression has no correlation with OS and PFS of patients with breast cancer (data not shown). With studies that reported that loss of ITM2A was a poor OS factor of cervical cancer (33), hepatocellular carcinoma (34), and acute myeloid leukemia (23), together with the fact that ITM2A has been observed to significantly decrease in those carcinoma types, we implied that ITM2A might be a novel tumor suppressor in those carcinomas that included breast cancer. Consistent to this point, our study demonstrated that upregulation of ITM2A reduced the aggressivity of breast cancer cells. Also, we found that ITM2A was correlated with immune response. Additionally, it was observed that ITM2A not only positively correlated with intensity of TILs and PD-L1 expression but also stimulated expression of PD-L1, PD-L2, and B7-H3 in breast cancer cells.

It is contentious to consider how critical the ITM2A is for the development of T effector cells (T effs). Tzong-Shyuan Tai et al. demonstrated that ITM2A plays a minimal role in development of T cells. On the other hand, other studies reported that overexpression of ITM2A in murine thymocytes resulted in a partial downregulation of CD8 in the CD4+CD8+ double positive thymocytes (35). What’s more, a study reported that ITM2A might be a susceptibility gene for graves’ disease (GD) in the Xq21.1 locus, strengthening the role of ITM2A in the immune system. This provoked our concentration on the association between ITM2A and tumor immunity in breast cancer. On the one hand, existing evidence demonstrated an association between ITM2A and immunity although this association was unclear. On the other hand, ITM2A is probably closely related to TILs and shapes the tumor microenvironment, which influences patients’ outcomes and treatment strategy once it is proved to play roles in T cells development. There are abundant studies showing that TILs can provide prognostic value for patients with breast cancer. For example, a pooled analysis of 3,771 patients treated with neoadjuvant therapy evaluated the correlation between TILs and prognosis in different subtypes of breast cancer. It was proved that increased TILs were associated with longer DFS in TNBC and HER2-positive breast cancer, longer OS in TNBC, and shorter OS in luminal B tumors (36). Our study demonstrated the positive correlation between ITM2A expression and TILs in breast cancer. Additionally, TILs and PD-L1 are helpful to choose patients who will receive more benefits from anti-PD-1/PD-L1 therapy. Patients with breast cancer that has over 1% cells stained with anti-PD-L1 antibody have higher rate of pRC than patients who expressed less than 1% anti-PD-L1 stained cells (8). Thus, we evaluated the relevance between ITM2A and TILs and PD-L1 expression based on TIMER. Importantly, we confirmed that ITM2A could stimulate PD-L1 expression in breast cancer cells. Collectively, high expression of ITM2A in breast cancer was accompanied with high intensity of TILs and abundant PD-L1 expression. This work provokes further study about predictive value of ITM2A in patients when they receive ICB treatments.

There are many limits in our study. We evaluated the correlations between ITM2A expression and TILs basing on a single database without verification. We did not explore the mechanism that ITM2A overexpression upregulates the PD-L1 expression. Those questions remain to be answered in further study.

## Conclusion

In summary, we found that ITM2A played a tumor suppressor role in breast cancer aggressivity, and had favorable effects on outcomes of patients with breast cancer. Meanwhile, ITM2A induced PD-L1 expression in breast cancer cells while accompanied with higher TILs numbers in the tumor microenvironment.

## Data Availability Statement

The original contributions presented in the study are included in the article/**Supplementary Material**. Further inquiries can be directed to the corresponding author.

## Ethics Statement

The studies involving human participants were reviewed and approved by the Ethics Committee of Tongji Hospital. The patients/participants provided their written informed consent to participate in this study. All animal procedures were performed in accordance with the approved Guide for the Care and Treatment of Laboratory Animals of Tongji Hospital and approved by the Ethics Committees of Tongji Hospital.

## Author Contributions

WC designed the experiments and supervised the study. RZ and TX performed the experiments. TX and RZ collected, analyzed, and interpreted the data. YX, ZW, and XL participated in revising the manuscript. All authors contributed to the article and approved the submitted version.

## Funding

This work was supported by the National Natural Science Foundation of China (81902933).

## Conflict of Interest

The authors declare that the research was conducted in the absence of any commercial or financial relationships that could be construed as a potential conflict of interest.

## Supplementary Material

Supplementary Figure 1 ITM2A expression levels in different cancers. ITM2A expression levels in different human cancers from TCGA database were shown by TIMER database. **p < 0.01, ***p < 0.001. TCGA, the Cancer Genome Atlas; TIMER, the Tumor Immune Estimation Resource.

Supplementary Figure 2 DEGs between MCF-7 cells that overexpressed ITM2A and expressed ITM2A normally. Volcano plot shows the 511 up-regulated genes (red) and 190 down-regulated genes (green) in ITM2A overexpression MCF-7 cells **(A)**. Heatmap of DEGs **(B)**.

Supplementary Figure 3 GSEA analysis implied ITM2A was active in immunity related response. GSEA was performed on RNA-Seq profiles of 1,053 breast cancer stratified by ITM2A mRNA expression levels and the top-10 ranked KEGG pathways were showed. GSEA, Gene Set Enrichment Analysis.

Supplementary Figure 4 ITM2A increased PD-L2 and B7-H3 expression in breast cancer cells. MCF-7 and MDA-MB-231 cells were transfected with indicated plasmid. PD-L2 **(A, B)** and B7-H3 **(C, D)** expression in these cells 48 hours after transfection were tested by flow cytometric analysis. ***p < 0.001.

Supplementary Figure 5 ITM2A expression was positively correlated with TILs quantity across common cancers. TIMER database was used to assess the correlation between ITM2A expression and six types of TILs enrichment cross five common cancers. TIMER analysis in lung adenocarcinoma **(A)**, lung squamous cell carcinoma **(B)**, colon adenocarcinoma **(C)**, head and neck cancer **(D)**, and prostate adenocarcinoma **(E)**. TIMER, the Tumor Immune Estimation Resource; TILs, tumor infiltration lymphocytes.

## Abbreviations

DMFS, Distant Metastasis Free Survival; RFS, Relapse Free Survival; OS, Overall Survival; DSS, Disease Specific Survival.

## References

[1] Hedlund J Johansson J Persson B. BRICHOS - a superfamily of multidomain proteins with diverse functions. BMC Res Notes (2009) 2:180. 10.1186/1756-0500-2-180 19747390 PMC2751770

[2] Tai T-S Pai S-Y Ho I-C. Itm2a, a Target Gene of GATA-3, Plays a Minimal Role in Regulating the Development and Function of T Cells. PloS One (2014) 9:e96535. 10.1371/journal.pone.0096535 24831988 PMC4022677

[3] American Cancer Society. Breast Cancer Facts & Figures. Am Cancer Soc (2021). Available at: https://www.cancer.org/research/cancer-facts-statistics/.

[4] Graham P. Metastatic breast cancer. Orthop Nurs (2018) 37:320–2. 10.1097/NOR.0000000000000494 30247418

[5] Bianchini G Balko JM Mayer IA Sanders ME Gianni L. Triple-negative breast cancer: challenges and opportunities of a heterogeneous disease. Nat Rev Clin Oncol (2016) 13:674–90. 10.1038/nrclinonc.2016.66 PMC5461122 27184417

[6] Solinas C Gombos A Latifyan S Piccart-Gebhart M Kok M Buisseret L. Targeting immune checkpoints in breast cancer: An update of early results. ESMO Open (2017) 2:e000255. 10.1136/esmoopen-2017-000255 29177095 PMC5687552

[7] Nanda R Liu MC Yau C Shatsky R Pusztai L Wallace A. Effect of Pembrolizumab Plus Neoadjuvant Chemotherapy on Pathologic Complete Response in Women With Early-Stage Breast Cancer. JAMA Oncol (2020) 6:676. 10.1001/jamaoncol.2019.6650 32053137 PMC7058271

[8] Adams S Loi S Toppmeyer D Cescon DW De Laurentiis M Nanda R. Pembrolizumab monotherapy for previously untreated, PD-L1-positive, metastatic triple-negative breast cancer: cohort B of the phase II KEYNOTE-086 study. Ann Oncol (2019) 30:405–11. 10.1093/annonc/mdy518 30475947

[9] Adams S Schmid P Rugo HS Winer EP Loirat D Awada A. Pembrolizumab monotherapy for previously treated metastatic triple-negative breast cancer: cohort A of the phase II KEYNOTE-086 study. Ann Oncol (2019) 30:397–404. 10.1093/annonc/mdy517 30475950

[10] Schmid P Cortes J Pusztai L McArthur H Kümmel S Bergh J. Pembrolizumab for Early Triple-Negative Breast Cancer. N Engl J Med (2020) 382:810–21. 10.1056/NEJMoa1910549 32101663

[11] Topalian SL Taube JM Anders RA Pardoll DM. Mechanism-driven biomarkers to guide immune checkpoint blockade in cancer therapy. Nat Rev Cancer (2016) 16:275–87. 10.1038/nrc.2016.36 PMC5381938 27079802

[12] Tumeh PC Harview CL Yearley JH Shintaku IP Taylor EJM Robert L. PD-1 blockade induces responses by inhibiting adaptive immune resistance. Nature (2014) 515:568–71. 10.1038/nature13954 PMC4246418 25428505

[13] Brahmer JR Drake CG Wollner I Powderly JD Picus J Sharfman WH. Phase I Study of Single-Agent Anti–Programmed Death-1 (MDX-1106) in Refractory Solid Tumors: Safety, Clinical Activity, Pharmacodynamics, and Immunologic Correlates. J Clin Oncol (2010) 28:3167–75. 10.1200/JCO.2009.26.7609 PMC4834717 20516446

[14] Cimino-Mathews A Thompson E Taube JM Ye X Lu Y Meeker A. PD-L1 (B7-H1) expression and the immune tumor microenvironment in primary and metastatic breast carcinomas. Hum Pathol (2016) 47:52–63. 10.1016/j.humpath.2015.09.003 26527522 PMC4778421

[15] Schalper KA Velcheti V Carvajal D Wimberly H Brown J Pusztai L. In Situ Tumor PD-L1 mRNA Expression Is Associated with Increased TILs and Better Outcome in Breast Carcinomas. Clin Cancer Res (2014) 20:2773–82. 10.1158/1078-0432.CCR-13-2702 24647569

[16] Chen B Lai J Dai D Chen R Li X Liao N. *JAK1* as a prognostic marker and its correlation with immune infiltrates in breast cancer. Aging (Albany NY) (2019) 11:11124–35. 10.18632/aging.102514 PMC6932910 31790361

[17] Nagy Á Lánczky A Menyhárt O Győrffy B. Validation of miRNA prognostic power in hepatocellular carcinoma using expression data of independent datasets. Sci Rep (2018) 8:9227. 10.1038/s41598-018-27521-y 29907753 PMC6003936

[18] Zheng H Zhang G Zhang L Wang Q Li H Han Y. Comprehensive Review of Web Servers and Bioinformatics Tools for Cancer Prognosis Analysis. Front Oncol (2020) 10:68. 10.3389/fonc.2020.00068 32117725 PMC7013087

[19] Livak KJ Schmittgen TD. Analysis of relative gene expression data using real-time quantitative PCR and the 2-ΔΔCT method. Methods (2001) 25:402–8. 10.1006/meth.2001.1262 11846609

[20] Subramanian A Tamayo P Mootha VK Mukherjee S Ebert BL Gillette MA. Gene set enrichment analysis: A knowledge-based approach for interpreting genome-wide expression profiles. Proc Natl Acad Sci (2005) 102:15545–50. 10.1073/pnas.0506580102 PMC1239896 16199517

[21] Zhang Y Tseng JT-C Lien I-C Li F Wu W Li H. mRNAsi Index: Machine Learning in Mining Lung Adenocarcinoma Stem Cell Biomarkers. Genes (Basel) (2020) 11:257. 10.3390/genes11030257 PMC7140876 32121037

[22] Zhou C Wang M Yang J Xiong H Wang Y Tang J. Integral membrane protein 2A inhibits cell growth in human breast cancer via enhancing autophagy induction. Cell Commun Signal (2019) 17:105. 10.1186/s12964-019-0422-7 31438969 PMC6704577

[23] Nagy Á Ősz Á Budczies J Krizsán S Szombath G Demeter J. Elevated HOX gene expression in acute myeloid leukemia is associated with NPM1 mutations and poor survival. J Adv Res (2019) 20:105–16. 10.1016/j.jare.2019.05.006 PMC6614546 31333881

[24] Zhao M Huang W Zou S Shen Q Zhu X. A Five-Genes-Based Prognostic Signature for Cervical Cancer Overall Survival Prediction. Int J Genomics (2020) 2020:1–13. 10.1155/2020/8347639 PMC7136791 32300605

[25] Nguyen TMH Shin I-W Lee TJ Park J Kim JH Park MS. Loss of ITM2A, a novel tumor suppressor of ovarian cancer through G2/M cell cycle arrest, is a poor prognostic factor of epithelial ovarian cancer. Gynecol Oncol (2016) 140:545–53. 10.1016/j.ygyno.2015.12.006 26691219

[26] Mizuno H Kitada K Nakai K Sarai A. PrognoScan: A new database for meta-analysis of the prognostic value of genes. BMC Med Genomics (2009) 2:18. 10.1186/1755-8794-2-18 19393097 PMC2689870

[27] Bradley JR. TNF-mediated inflammatory disease. J Pathol (2008) 214:149–60. 10.1002/path.2287 18161752

[28] Ouyang W Rutz S Crellin NK Valdez PA Hymowitz SG. Regulation and functions of the IL-10 family of cytokines in inflammation and disease. Annu Rev Immunol (2011) 29:71–109. 10.1146/annurev-immunol-031210-101312 21166540

[29] Sun C Mezzadra R Schumacher TN. Regulation and Function of the PD-L1 Checkpoint. Immunity (2018) 48:434–52. 10.1016/j.immuni.2018.03.014 PMC7116507 29562194

[30] Taube JM Klein A Brahmer JR Xu H Pan X Kim JH. Association of PD-1, PD-1 Ligands, and Other Features of the Tumor Immune Microenvironment with Response to Anti-PD-1 Therapy. Clin Cancer Res (2014) 20:5064–74. 10.1158/1078-0432.CCR-13-3271 PMC4185001 24714771

[31] Loi S Sirtaine N Piette F Salgado R Viale G Van Eenoo F. Prognostic and Predictive Value of Tumor-Infiltrating Lymphocytes in a Phase III Randomized Adjuvant Breast Cancer Trial in Node-Positive Breast Cancer Comparing the Addition of Docetaxel to Doxorubicin With Doxorubicin-Based Chemotherapy: BIG 02-98. J Clin Oncol (2013) 31:860–7. 10.1200/JCO.2011.41.0902 23341518

[32] Savas P Salgado R Denkert C Sotiriou C Darcy PK Smyth MJ. Clinical relevance of host immunity in breast cancer: from TILs to the clinic. Nat Rev Clin Oncol (2016) 13:228–41. 10.1038/nrclinonc.2015.215 26667975

[33] Meng H Liu J Qiu J Nie S Jiang Y Wan Y. Identification of Key Genes in Association with Progression and Prognosis in Cervical Squamous Cell Carcinoma. DNA Cell Biol (2020) 39:848–63. 10.1089/dna.2019.5202 32202912

[34] Zhang Z Li J He T Ouyang Y Huang Y Liu Q. The competitive endogenous RNA regulatory network reveals potential prognostic biomarkers for overall survival in hepatocellular carcinoma. Cancer Sci (2019) 110:2905–23. 10.1111/cas.14138 PMC6726690 31335995

[35] Kirchner J Bevan MJ. Itm2a Is Induced during Thymocyte Selection and T Cell Activation and Causes Downregulation of Cd8 When Overexpressed in Cd4+Cd8+ Double Positive Thymocytes. J Exp Med (1999) 190:217–28. 10.1084/jem.190.2.217 PMC2195576 10432285

[36] Denkert C von Minckwitz G Darb-Esfahani S Lederer B Heppner BI Weber KE. Tumour-infiltrating lymphocytes and prognosis in different subtypes of breast cancer: a pooled analysis of 3771 patients treated with neoadjuvant therapy. Lancet Oncol (2018) 19:40–50. 10.1016/S1470-2045(17)30904-X 29233559

