# Supplementary figures and images for: ITM2A as a Tumor Suppressor and Its Correlation With PD-L1 in Breast Cancer
Source: Front Oncol. 2021 Feb 12;10:581733. doi: 10.3389/fonc.2020.581733 (PMC7928367; doi:10.3389/fonc.2020.581733)

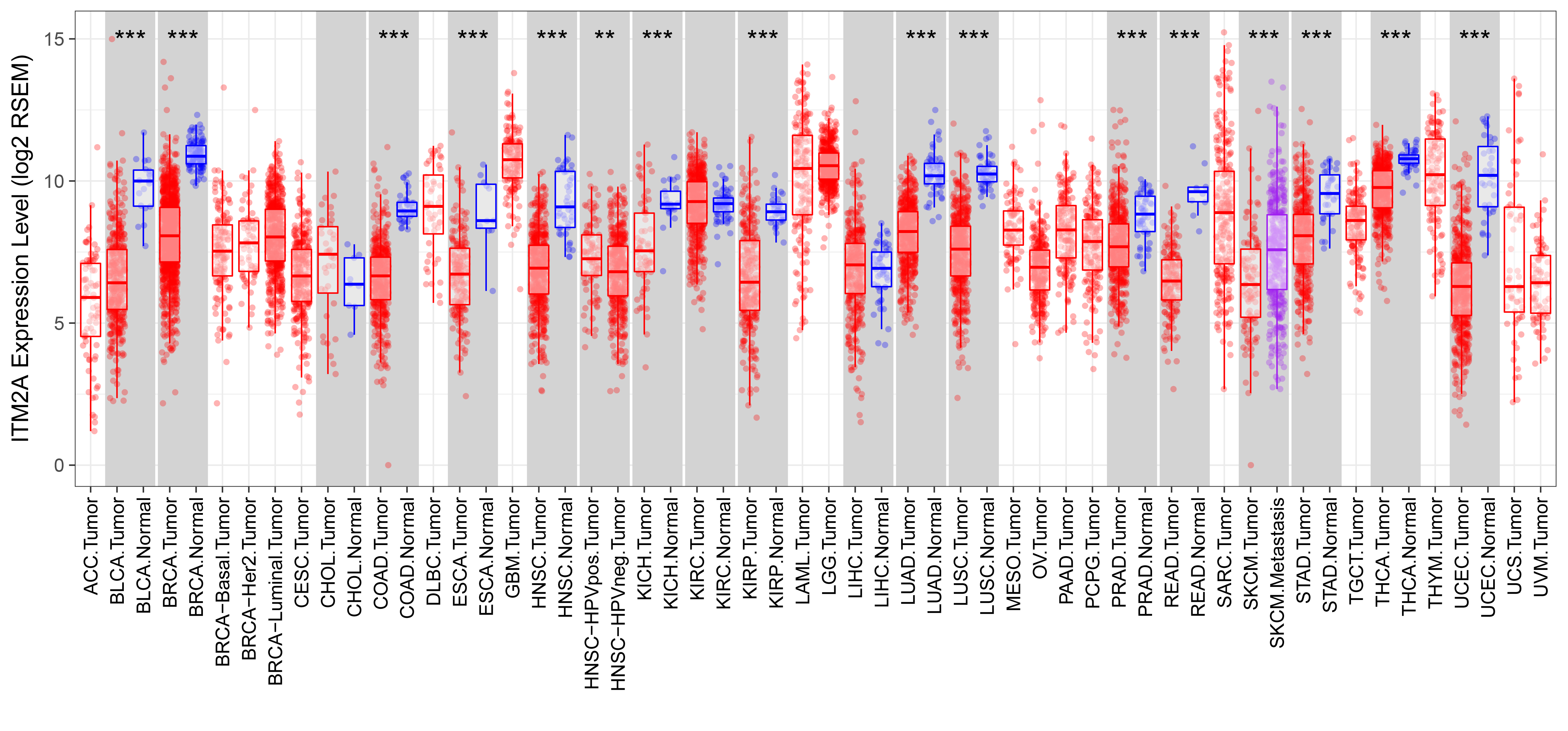

Supplement: Supplementary Figure 1 — ITM2A expression levels in different cancers. ITM2A expression levels in different human cancers from TCGA database were shown by TIMER database. **p < 0.01, ***p < 0.001. TCGA, the Cancer Genome Atlas; TIMER, the Tumor Immune Estimation Resource. [file Image_1.tif]

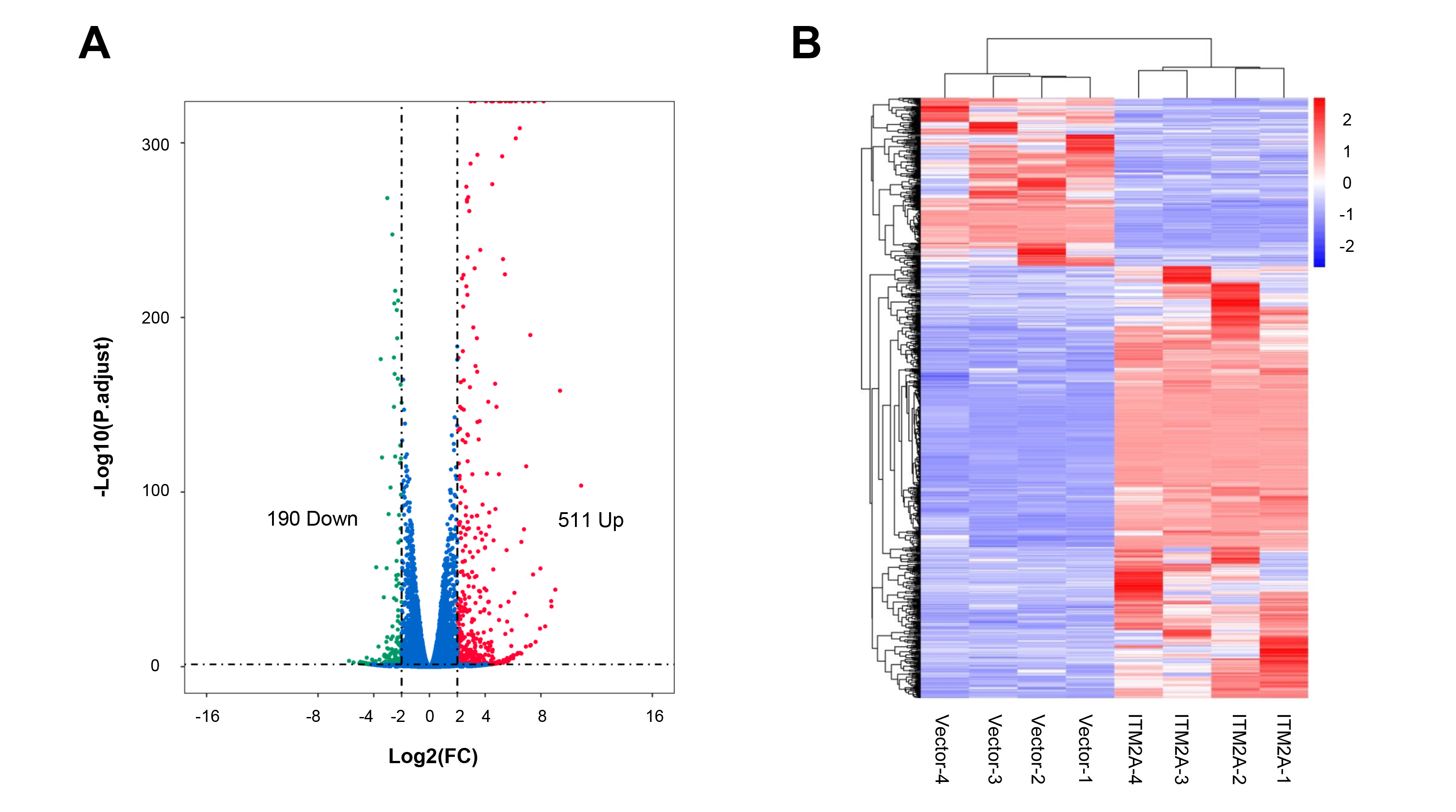

Supplement: Supplementary Figure 2 — DEGs between MCF-7 cells that overexpressed ITM2A and expressed ITM2A normally. Volcano plot shows the 511 up-regulated genes (red) and 190 down-regulated genes (green) in ITM2A overexpression MCF-7 cells (A). Heatmap of DEGs (B). [file Image_2.tif]

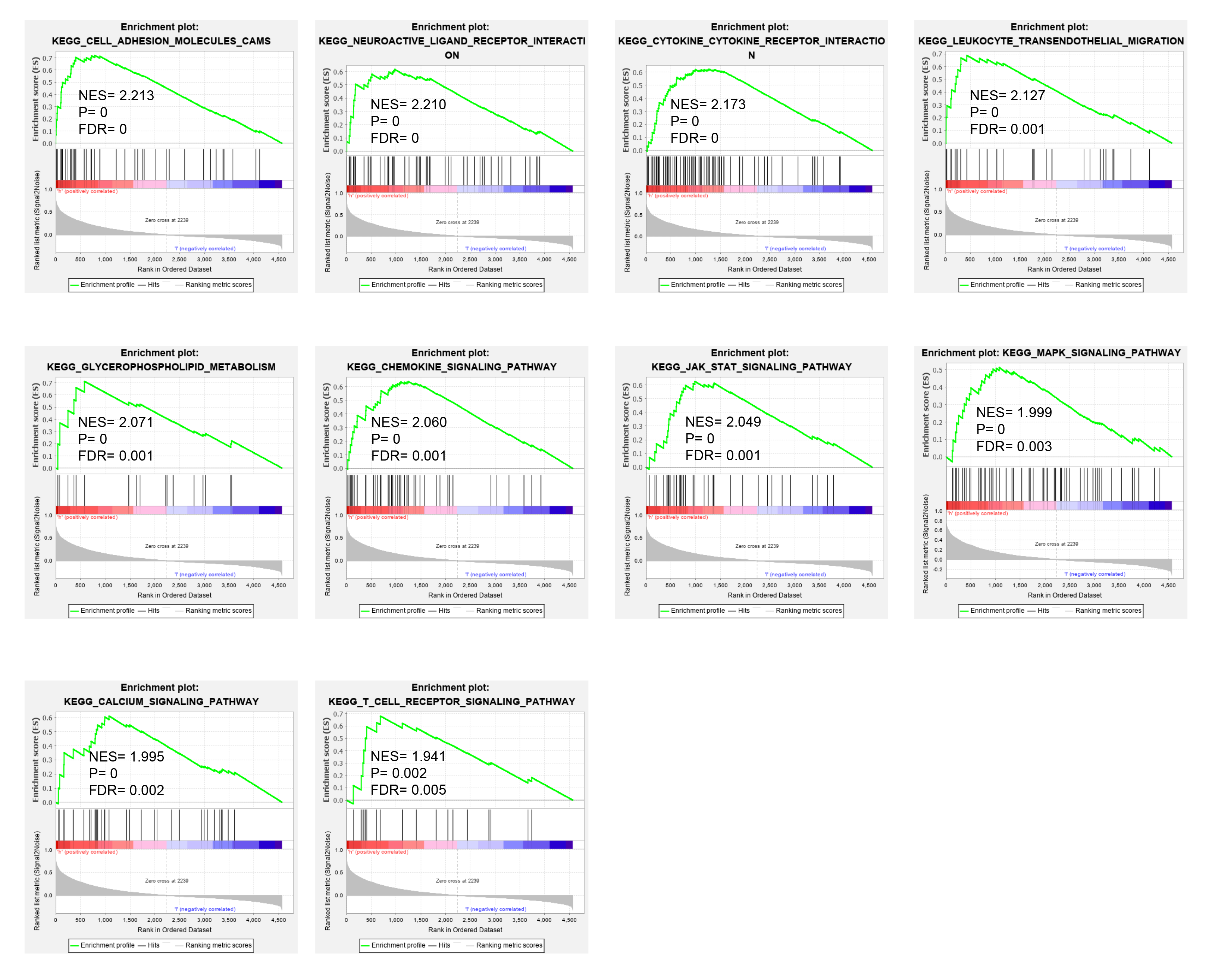

Supplement: Supplementary Figure 3 — GSEA analysis implied ITM2A was active in immunity related response. GSEA was performed on RNA-Seq profiles of 1,053 breast cancer stratified by ITM2A mRNA expression levels and the top-10 ranked KEGG pathways were showed. GSEA, Gene Set Enrichment Analysis. [file Image_3.tif]

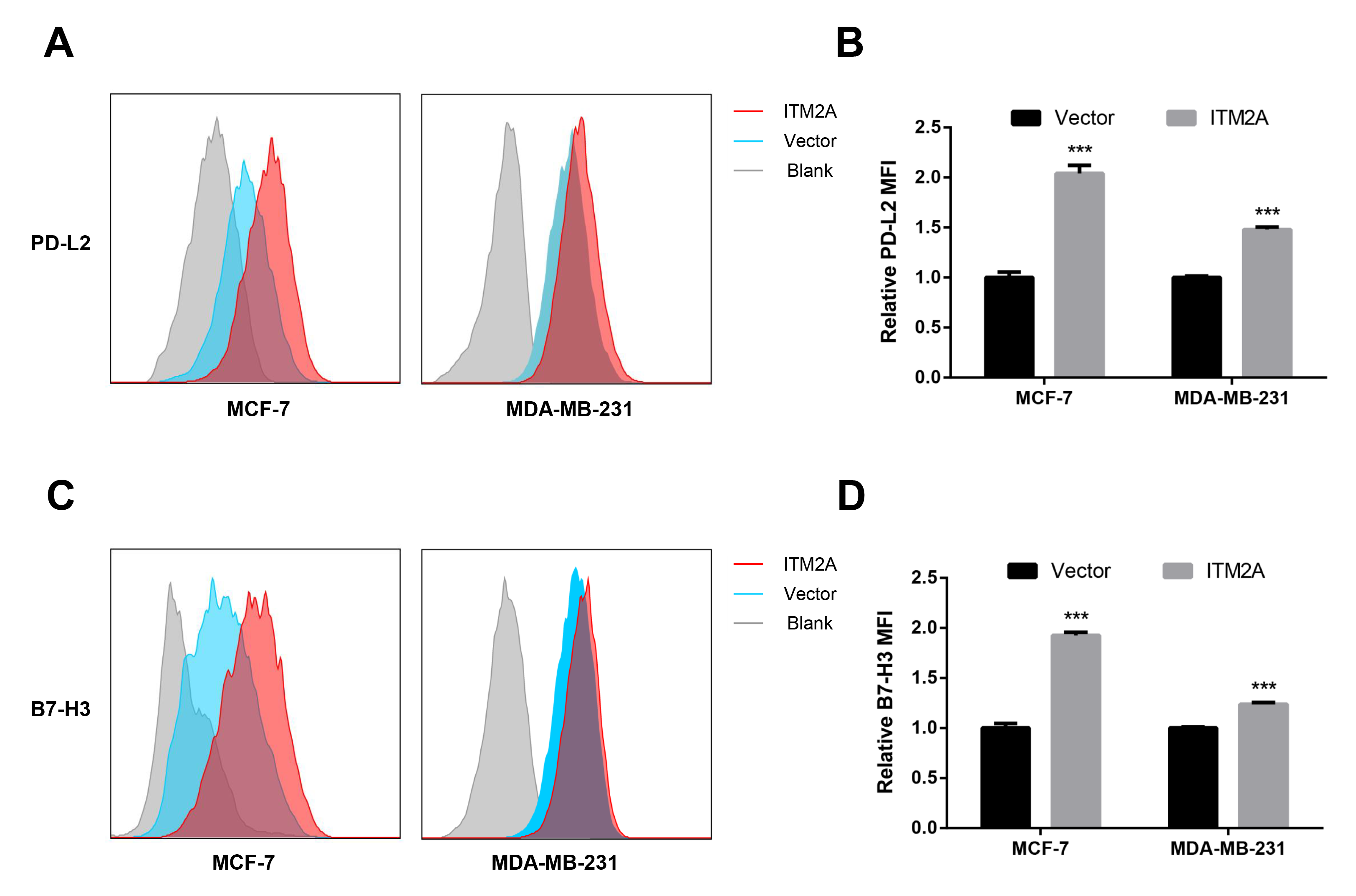

Supplement: Supplementary Figure 4 — ITM2A increased PD-L2 and B7-H3 expression in breast cancer cells. MCF-7 and MDA-MB-231 cells were transfected with indicated plasmid. PD-L2 (A, B) and B7-H3 (C, D) expression in these cells 48 hours after transfection were tested by flow cytometric analysis. ***p < 0.001. [file Image_4.tif]

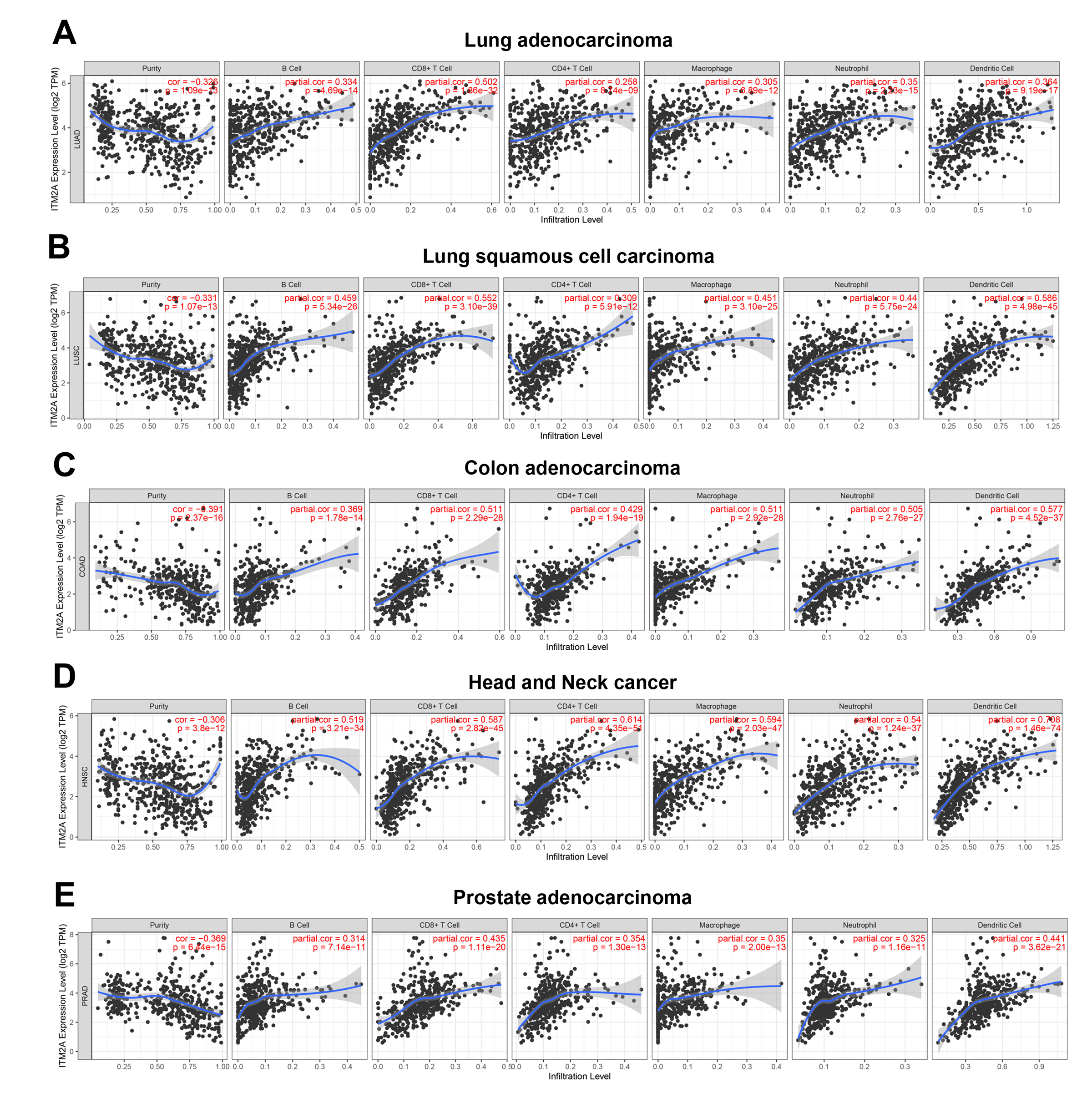

Supplement: Supplementary Figure 5 — ITM2A expression was positively correlated with TILs quantity across common cancers. TIMER database was used to assess the correlation between ITM2A expression and six types of TILs enrichment cross five common cancers. TIMER analysis in lung adenocarcinoma (A), lung squamous cell carcinoma (B), colon adenocarcinoma (C), head and neck cancer (D), and prostate adenocarcinoma (E). TIMER, the Tumor Immune Estimation Resource; TILs, tumor infiltration lymphocytes. [file Image_5.tif]
